# MEMS Skin Friction Sensor with High Response Frequency and Large Measurement Range

**DOI:** 10.3390/mi13020234

**Published:** 2022-01-30

**Authors:** Huihui Guo, Xiong Wang, Tingting Liu, Zhijiang Guo, Yang Gao

**Affiliations:** 1 School of Information Engineering, Southwest University of Science and Technology, Mianyang 621010, China; easonguo@swust.edu.cn (H.G.); guozhijiang@swust.edu.cn (Z.G.); gaoy@swust.edu.cn (Y.G.); 2 Robot Technology Used for Special Environment Key Laboratory of Sichuan Province, Mianyang 621010, China; 3 Hypervelocity Aerodynamics Institute, China Aerodynamics Research and Development Center, Mianyang 621010, China

**Keywords:** skin friction sensors, shock tunnel experiment, MEMS, fast readout circuit

## Abstract

Micro-electromechanical system (MEMS) skin friction sensors are considered to be promising sensors in hypersonic wind tunnel experiments owing to their miniature size, high sensitivity, and stability. Aiming at the problem of short test duration (a few milliseconds) and heavy load in a shock wind tunnel, the fast readout circuit and the sensor head structures of a MEMS skin friction sensor are presented and optimized in this work. The sensor was fabricated using various micro-mechanical processes and micro-assembly technology based on visual alignment. Meanwhile, the sensor head structure was integrated with the fast readout circuit and tested by using a centrifugal force equivalent method. The calibration results show that this sensor provides good linearity, sensitivity, and stability. The measurement ranges are 0–2000 Pa with good performance. The resolution is better than 10 Pa at 3000 Hz detection frequency of the readout circuit for the sensor in ranges from 0 to 1000 Pa. In addition, the repeatability and linearity of static calibration for sensors are better than 1%.

## 1. Introduction

The skin friction resistance of an aircraft surface is an important part of its total resistance, which greatly limits the performance of hypersonic vehicles. Therefore, the skin friction measurement of an aircraft model is an important basic item in aerodynamic research. MEMS skin friction sensors are considered promising sensors in hypersonic wind tunnel experiments owing to their miniature size, high sensitivity, and stability. In recent years, several researchers have developed MEMS sensors to measure skin friction, including the capacitance-type and comb differential capacitance-type [1,2,3,4,5], piezoresistive-type [6,7,8], and piezoelectric-type [9]. For example, Mills et al. [5] reported a fully differential capacitive wall shear stress sensor for low-speed wind tunnels with the high sensitivity of 196 mV/Pa and a minimum detection limit of 12 mPa at 1000 Hz in a range from 0–10 Pa; Von, P. et al. [8] reported a wall shear stress sensor using four piezoresistors in the cantilever, and the resolution was 0.01 Pa in the range of 2 Pa; Kim, T. et al. [9] reported a piezoelectric floating element shear stress sensor for the wind tunnel flow measurement with the high sensitivity of 56.5 pc/Pa and with the frequency range of the sensor up to 800 Hz. The common characteristics of those sensors are their usually high sensitivity, high resolution (10^−2^ Pa), and small measurement range (several Pa). Therefore, these sensors are mainly used in low wind tunnels to measure skin friction because their sensing elements, such as the comb capacitance, were exposed in the flow field. To adapt to the harsh measurement environments and large normal loads in hypersonic fields, a novel MEMS skin sensor was developed in our previous work [10,11]. The sensor adopts the floating element so that it is even with the measured wall, and the signal output micro-structure is isolated from the hypersonic field, which was fabricated using various micro-mechanical processes and micro-assembly technology. Experimental results show that this sensor has some good characteristics, such as good linearity, small size, high sensitivity, and repeatability of static calibration. Furthermore, the measurement ranges were 0–100 Pa, and the minimum detectable skin friction was 0.1 Pa. However, the natural frequency of the sensor was 410 Hz, and the detection frequency of a weak capacitance readout circuit for a skin friction sensor is only 10 Hz.

According to the hypersonic flow field characteristics of high temperature, high total pressure, and short test time in the shock wind tunnel, the test ambient temperature is usually increased to hundreds of degrees centigrade, and the measurement ranges increase to hundreds, or even thousands, of Pa. Meanwhile, the sampling frequency of the readout circuit for the sensor also increases to thousands of Hz with higher loads, and only several milliseconds in duration of the working time. Therefore, greater measurement ranges and higher response frequencies of sensors for skin friction in shock wind tunnels need to be developed.

In this work, the static force analysis and modal analysis of the sensor head structure were carried out by finite element analysis (FEA) to optimize the head structure of the MEMS skin friction sensor with a high response frequency and large measurement ranges. In addition, to improve the detection frequency of the weak capacitance of the sensor, the fast readout circuit with 3000 Hz detection frequency was designed and integrated with the sensor head structure. 

## 2. Structural Simulation and Optimization of the Sensor Head Structure

**Figure 1:**
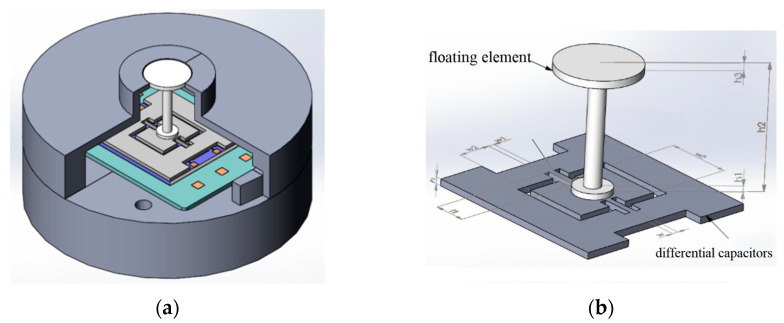
(**a**) Schematic of the sensor; (**b**) the sensing structure parameters of the sensor head.

The structure of the MEMS skin friction sensor is shown in Figure 1. The sensor was composed of a silicon differential capacitor embedded with a floating element, a signal readout circuit, and a metal shell package. The working principle of this sensor is explained as the skin friction caused the torsional deflection of clamped-clamped elastic beams by the floating element, and the torsional deflection was transferred into a capacitance variation by one pair of differential capacitors.

For a typical shock tunnel with a short test time (a few milliseconds), the lowest frequency to obtain high measurement accuracy within the effective test time is 1000 Hz. Because the mechanical vibration of the measurement system is excited and cannot be attenuated rapidly enough by damping characteristics during the short test time, to obtain higher measurement accuracy, the natural frequency of the MEMS skin friction sensor should, therefore, be raised to 3000 Hz. 

For an undamped single freedom system, the natural frequency calculation formula is defined as shown in the following equation:

(1) $$f_{n}=\frac{1}{2\pi }\sqrt{\frac{k}{m}}$$

where $f_{n}$ is the natural frequency, and k and *m* are the stiffness and mass of the structure, respectively. From Equation (1), it can be seen that increasing the stiffness of the beam and reducing the mass of the floating element was an effective way to improve the natural frequency of a MEMS skin friction sensor. Then, the sensitivity of the sensor deteriorated rapidly because the torsional deflection of the elastic beams decreased with an increase in the stiffness (with increasing *w* and *h*) under the same loads. The sensitivity of the sensor can be defined according to conformal transformation theory [11]:

(2) $$S\approx \frac{\tau_{w}A}{\Delta C}=\frac{h_{{}_{0}}^{2}}{\varepsilon_{0}(2w_{1}+w_{2})w_{2}w_{3}}\times \frac{\beta }{1+\nu }\times \frac{Ew^{3}h}{l_{1}h_{2}}$$

where $\tau_{w}$ is the measured skin friction, ΔC is the variation of differential capacitance, A is the area of the floating element, β, ν, $\varepsilon_{0}$ and *E* are the elastic deformation parameters of the torsion beam, respectively, *w*, $l_{1}$ and h are the length width and thickness of the silicon beam, and $h_{0}$ and $h_{2}$ are the gaps of the capacitor and the height of the floating element as a force arm. To fabricate the high-performance skin friction sensor for a shock wind tunnel experiment, the sensitivity and the natural frequency were considered together. 

**Figure 2:**
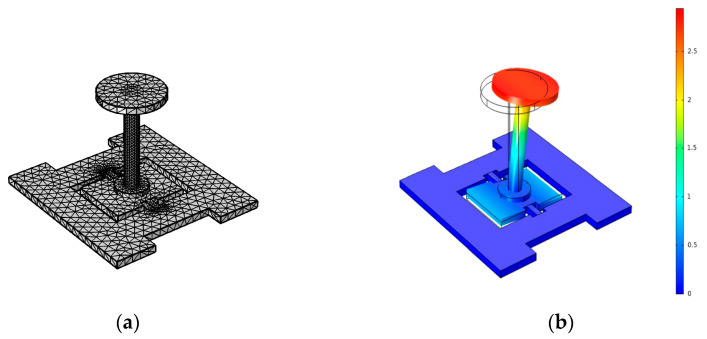
(**a**) Three-dimensional FEA model of the sensor head structure; (**b**) the normal deformation of the vibrating plate under 2000 Pa equivalent loads.

To obtain the main structural parameters of the silicon beam and the floating element, three-dimensional FEA models of the sensor head structures were built with the help of FEA software, using our previous parameters. The optimized three-dimensional FEA model of the sensor head structure is shown in Figure 2a. 

**Figure 3:**
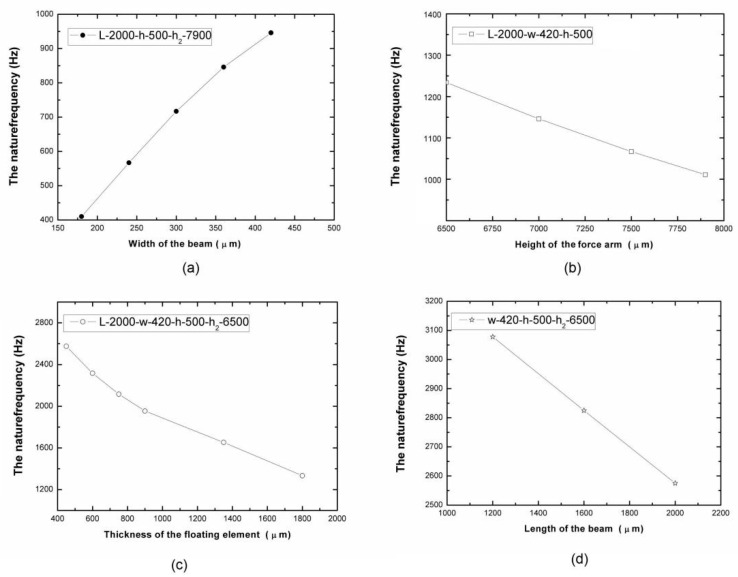
The variation of the natural frequency with different structure parameters of the sensor head: (**a**) the width changes of the silicon beam; (**b**) the height changes of the force arm; (**c**) the thickness changes of the floating element; (**d**) the length changes to the silicon beams.

Combining the designed indexes and our previous research results, we determined that increasing the width of the silicon beam was key to improving the stiffness of the sensor head structure. To ensure the working state of the torsion beam, the width was not allowed to exceed its thickness (the thickness of the wafer was about 500 μm). The natural frequency of the sensor head with different widths of the silicon beam is shown in Figure 3a. It can be seen that the natural frequency of the sensor head was from 410 Hz to 1000 Hz as the width of the silicon beam was increased from 180 μm to 420 μm. However, the natural frequency increase tended to slow down when the width of the silicon beam was increased to 420 μm. Meanwhile, the sensitivity of the sensor was down 90 percent by Equation (2). The maximum width of the beam can be determined as 420 μm without considering the sensitivity of the sensor in this work.

To further enhance the natural frequency, the height of the floating element as a force arm and the mass of the floating element were reduced. The frequency increased from 1000 Hz to 1250 Hz as the height of the force arm was decreased from 7900 μm to 6500 μm, as shown in Figure 3b. It can also be seen that reducing the force arm had little effect on improving the natural frequency of the sensor head. The reason is that the reduction of the length of the floating element has little effect on the overall quality of m. 

Therefore, reducing the thickness of the floating element can quickly reduce its overall mass to improve the natural frequency of the sensor, as shown in Figure 3c. However, the rapid reduction of the overall mass led to the rapid reduction of the equivalent torque and the sensitivity. Combining the influence rule of the skin friction measurements model [12,13] and the precision machining process, the thickness parameters of the floating element were determined as 450 μm in this work. 

The influence of the length of the beam on the natural frequency of the sensor was also simulated, as shown in Figure 3d. The simulation results show that the natural frequency of the sensor head increased linearly with the decreasing length of the beam.

Finally, to increase the natural frequency of the sensor to over 3000 Hz, the main structure parameters were achieved, as shown in Table 1. 

Meanwhile, the skin friction *τ_w_* was equivalent to the shear stress load on the surface of the floating element in the finite element simulation. The displacement of the differential capacitor plate under 2000 Pa loads is shown in Figure 2b. It can be seen that the maximum normal deformation of the edge of the vibrating plate away from the torsion beam was +1.06 μm and −1.05 μm, respectively. To ensure the good linearity of the sensor in the total measurement range, the gap of the differential capacitor was larger than 6 μm. In this work, the gap of the differential capacitor was designed as 10 μm, and the initial capacitance was about 9 pF by calculation. At the same time, the design sensitivity was about 2.5 Pa/fF in the range of 0–2000 Pa by Equation (2).

## 3. The Fast Readout Circuit Design

**Figure 4:**
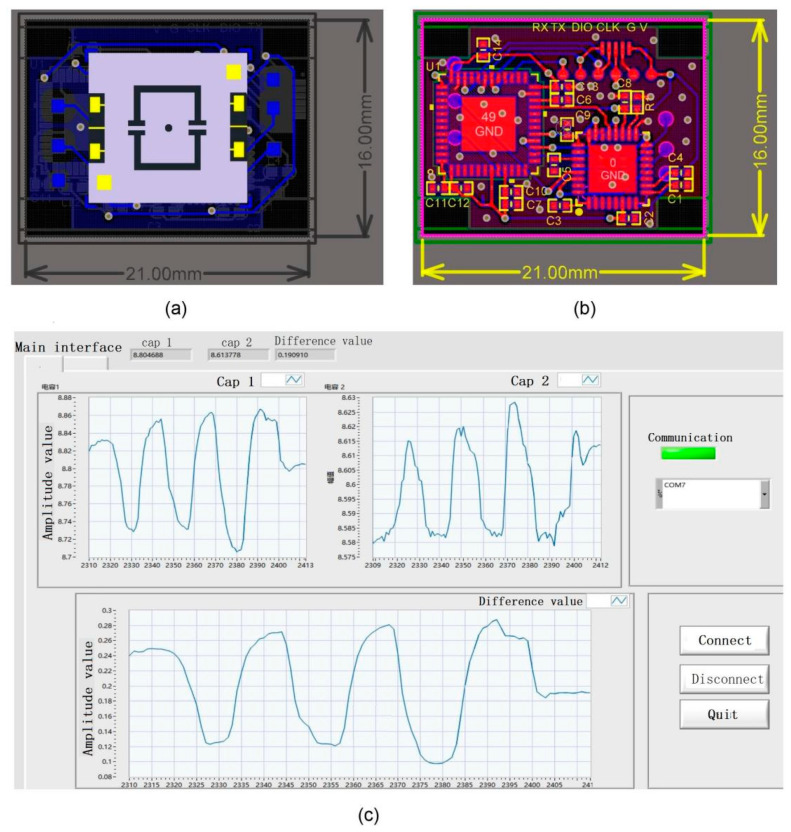
(**a**) Schematic of the readout circuit integrated with MEMS chip; (**b**) the PCB of the circuit; (**c**) the display functional interfaces for the MEMS skin friction sensor.

Considering the previous simulation and calculation results, the variation of the capacitance under the skin friction load was very small, within 0.01~1.00 pF. Thus, the circuit needs not only high measurement precision but also a high detection frequency for a shock tunnel with the short time of a few milliseconds. To improve the measurement precision, the signal wire between the sensor head and readout circuit were as short as possible, thus, the readout circuit was integrated with the MEMS chip, as shown in Figure 4a. The relationship between the detection frequency and the measurement accuracy of the sensor was contradictory. It was key to select a weak capacitance detection chip with a high detection frequency and measurement accuracy. The readout circuit included weak capacitance testing, and the processing circuit part and the software real-time display part were designed in this work. A Pcap01 chip is an application-specific integrated circuit for a MEMS capacitance sensor with a low-power capacitance-to-digital converter with a wide input range (fF to nF) and versatile configuration options, and it can be configured for the highest sampling rates of up to 500 kHz, the lowest current consumption of down to 2 uA, or the lowest noise of 15 aF (RMS). In this work, the capacitance signal testing and processing circuit were mainly composed of a Pcap01 chip and single chip (STM32F411CEU6) due to the limitation of the volume, as shown in Figure 4b, which was fabricated by a precision ceramic micro-strip circuit process. The real-time detection and display functional interfaces of the MEMS skin friction sensor were designed using LabVIEW software, as shown in Figure 4c. 

Although the measurement accuracy deteriorated with the increase in sampling frequency, the readout circuit for the MEMS skin sensor still had sufficiently high detection accuracy at 3000 Hz to meet the testing requirements in a shock wind tunnel. The resolution was about 10 Pa by static calibration test using a centrifugal force equivalent method. 

**Figure 5:**
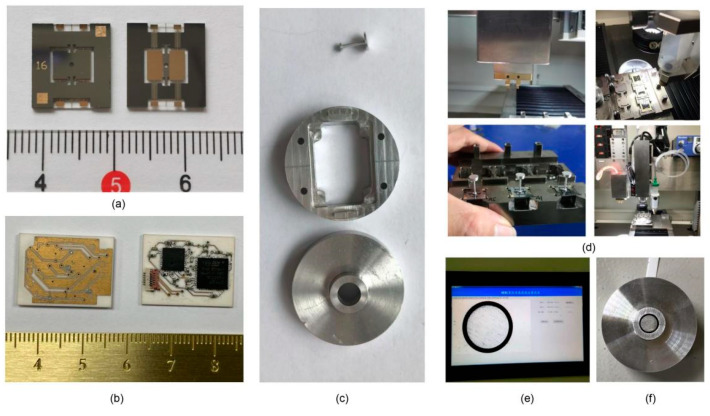
The photos of the separate components for MEMS sensor (**a**–**c**); (**d**) the micro-assembly processes for MEMS sensor; (**e**) the coaxiality measurement for sensor; (**f**) the photo of the assembled sensor.

The components of the MEMS skin friction sensors, including the MEMS chip, floating element, signal readout circuit, and package shell, were fabricated using MEMS and precision machining process technology. MEMS chips were fabricated using the MEMS process, which consists of a silicon structure and glass electrode substrate. The photo of a single chip is shown in Figure 5a. The signal readout circuit was fabricated by precise microstrip circuit technology to ensure the precision of the components and processing. The photo of the ceramic circuit board is shown in Figure 5b. The material of the floating elements and package shells was aluminum alloy to be consistent with the test model material. The precision turning and milling technologies with 10 μm geometrical precision were used to fabricate the floating element and package shells. A photo of the separate components is shown in Figure 5c.

The accuracy of the skin friction measurements is very closely related to the assembly error of the sensor and the flow characteristics of the measured wall [11]. To reduce assembly error, a micro-assembly method for MEMS skin friction sensor assembly based on visual alignment was developed in our previous work [10]. The key parts mainly included the floating element and MEMS chip assembly, the MEMS chip and readout circuit assembly, and the sensor head and package assembly, which are shown in Figure 5d. 

The assembly results show that the assembly error can be well controlled by using visual alignment and a micro-operating assembly system. The coaxiality of the MEMS skin friction sensor was very good, and the error was only 2 μm by calculating pixels, as shown in Figure 5e. A photo of the sensor with good electrical characteristics and small assembly error is shown in Figure 5f.

## 4. Static Calibration of the Sensor

**Figure 6:**
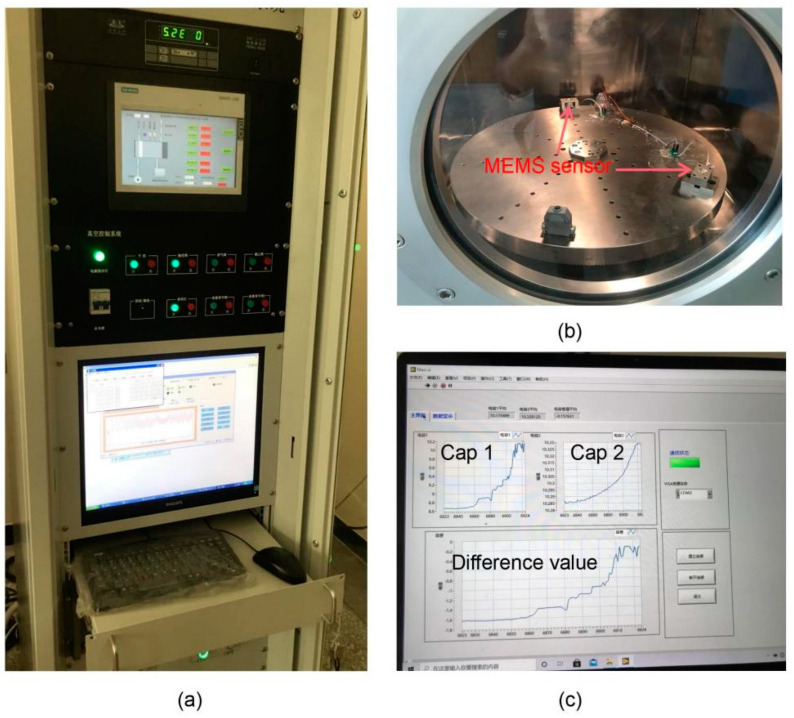
(**a**) Control system of the loading platform; (**b**) rotary loading platform in vacuum environment; (**c**) signal real-time detection, storage, and display window of the MEMS skin friction sensor.

The performances of the MEMS skin friction sensor were determined after assembly. With an experiment of static loading force, the static performance parameters of the sensor were obtained. Due to the small skin friction and volume of the MEMS sensor, the centrifugal force equivalent method and a single-spindle rotary loading platform were adopted to statically calibrate the sensor [11]. The static calibration system for the sensor is shown in Figure 6.

The MEMS sensors were fixed onto a turntable in a vacuum environment, as shown in Figure 6b. The skin friction sensed by the floating element was in some cases equivalent to the rotating speed of the turntable when the structure parameters of the sensor were determined. Then, the relationship between the equivalent friction and capacitance were obtained. The variation of capacitance under the equivalent friction loads was displayed and stored in real time, as shown in Figure 6c. 

**Figure 7:**
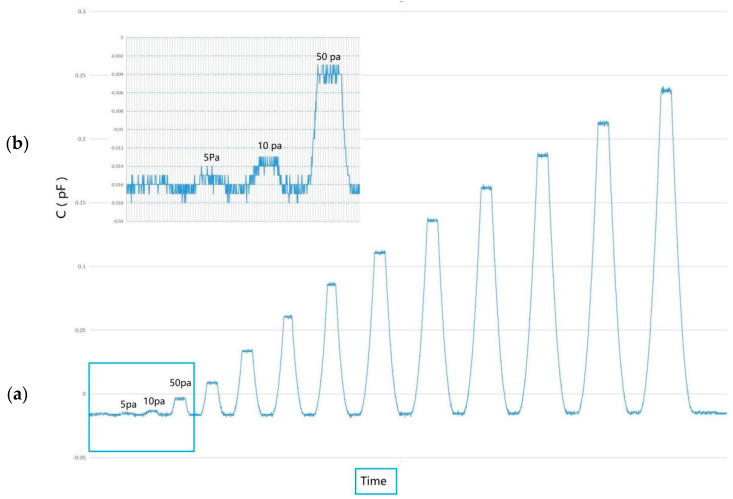
(**a**) The output response of the MEMS skin friction sensor under different equivalent loads; (**b**) the output response of the sensor under small loads.

The maximum equivalent load was set to 1000 Pa, which was limited by the rotating speed of the turntable. The output response of the MEMS skin friction sensor under different equivalent loads is shown in Figure 7. The detection limit of the sensors was determined by the head structure of the sensor and the noise of the readout circuit. The inset shows the responses of the sensor under small equivalent loads. It can be seen that the response curve displays a clear stepwise increase when the load was increased from 0 to 10 Pa. The resolution of the sensor was better than 10 Pa at the same time.

**Figure 8:**
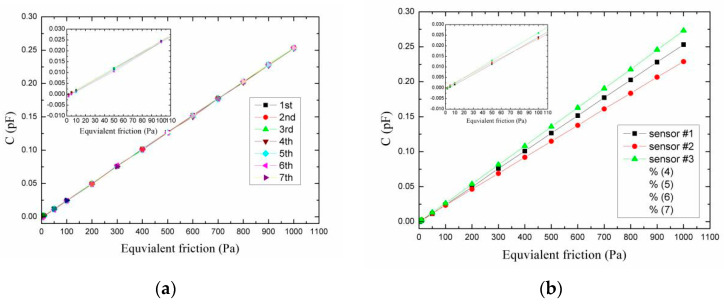
(**a**) The repeatability curves of sensor #1; (**b**) the response curves of three sensors under different loads.

To obtain the repeatability characteristics of the sensor, seven groups of response values under the same loading conditions were collected and stored at one time. To obtain the response curve of the sensor under different loads, the response values were averaged during a steady load. The repeatability curve of sensor #1 under different loads is shown in Figure 8a. It can be seen that the sensor has good linearity and stability. The linearity and repeatability are both better than 1% in the range from 0 to 1000 Pa, the sensitivity is about 3.8 Pa/fF, and the detection limit is about 10 Pa. In addition, the inset of Figure 8a shows the responses of the sensor in a range up to 100 Pa. 

Although all components of the sensors were manufactured in the same batch, the machining errors and assembly errors still affected the consistency of the sensors. The response characteristics of the three sensors are shown in Figure 8b. Those sensors also had good linearity and stability in the range of 0 to 1000 Pa. The sensitivities were 3.8 Pa/fF, 4.3 Pa/fF, and 3.6 Pa/fF, respectively, and the resolution was better than 10 Pa, which met the needs of the shock wind tunnel experiment. 

## 5. Conclusions

This paper developed a MEMS skin friction sensor with high detection frequency and a large measurement range in a shock wind tunnel experiment. With the help of finite element analysis software, the sensor head structure of an MEMS sensor was optimized to enhance the natural frequency to 3000 Hz. A key factor was to quickly increase the natural frequency of the sensor head with coarse-tuning by increasing the width of the beam and reducing the mass of the floating element. Then, the length of the torsional beam and the floating element as the force arm was used to achieve fine-tuning. However, the sensitivity deteriorated rapidly with the increasing natural frequency. In addition, to meet the testing requirement in the shock wind tunnel with a short test duration (a few milliseconds), it was key to design a weak capacitance detection circuit with high detection frequency and measurement accuracy. The readout circuit of the sensor was designed and integrated with the sensor head. The components of the sensor, including the MEMS chip, floating element, signal readout circuit, and package shell, were fabricated by using various micro-mechanical processes.

The sensors were assembled using micro-assembly technology based on visual alignment with small assembly errors. The coaxiality of the MEMS skin friction sensor was very good, and the error was only 2 μm. This is the main reason for the good manufacturing consistency of the sensor.

Then, the static performance parameters of the sensor were obtained using the centrifugal force equivalent method and the single-spindle rotary loading platform. The calibration results show that the sensors have good linearity and stability in a range from 0 to 1000 Pa. The repeatability and linearity of static calibration for sensors are better than 1%, and the resolution is better than 10 Pa at the 3000 Hz detection frequency of the sensor. Due to the maximum deformation values of the vibrating plate under 2000 Pa being only 10 percent of the differential capacitor gap, the sensor will have good linearity in a large range (greater than 2000 Pa). Moreover, the gap of the differential capacitor is also one of the important parameters affecting the sensitivity of the sensor. The reduction of the capacitance gap is a key factor to quickly enhance the sensitivity of the sensor. For example, the sensitivity will double with the decrease of the gap of the capacitor from 10 μm to 7 μm in future work.

## Figures and Tables

**Table 1:** The main structural parameters of the sensor.

Width of Silicon Beam (μm)	Length of Silicon Beam (μm)	The Thickness of the Silicon Beam (μm)	Diameter of the Floating Element (μm)	Thickness of Floating Element (μm)	Height of Floating Element (μm)	Gap of Capacitor (μm)
400	1200	500	5000	450	7000	10

## Data Availability

Data are contained within the article.
